# EphB2 signaling regulates lesion-induced axon sprouting but not critical period length in the postnatal auditory brainstem

**DOI:** 10.1186/1749-8104-8-2

**Published:** 2013-02-05

**Authors:** Paul A Nakamura, Karina S Cramer

**Affiliations:** 1 Department of Neurobiology and Behavior and Center for the Neurobiology of Learning and Memory, University of California, Irvine, CA, 92697, USA

**Keywords:** MNTB, auditory, brainstem, axon guidance, deafferentation, regeneration

## Abstract

**Background:**

Studies of developmental plasticity may provide insight into plasticity during adulthood, when neural circuitry is less responsive to losses or changes in input. In the mammalian auditory brainstem, globular bushy cell axons of the ventral cochlear nucleus (VCN) innervate the contralateral medial nucleus of the trapezoid body (MNTB) principal neurons. VCN axonal terminations in MNTB, known as calyces of Held, are very large and specialized for high-fidelity transmission of auditory information. Following unilateral deafferentation during postnatal development, VCN axons from the intact side form connections with novel targets, including the ipsilateral MNTB. EphB signaling has been shown to play a role in this process during the first postnatal week, but mechanisms involved in this reorganization during later developmental periods remain unknown.

**Results:**

We found that EphB2 signaling reduces the number of induced ipsilateral projections to the MNTB after unilateral VCN removal at postnatal day seven (P7), but not after removal of the VCN on one side at P10, after the closure of the critical period for lesion-induced innervation of the ipsilateral MNTB.

**Conclusions:**

Results from this study indicate that molecular mechanisms involved in the development of circuitry may also play a part in rewiring after deafferentation during development, but do not appear to regulate the length of critical periods for plasticity.

## Background

Development marks a period of remarkable plasticity and circuit malleability. Neural circuitry is most susceptible to changes in input during developmental critical periods when connections display the most profound functional and structural responses to changes in input [1-8]. Axons of globular bushy cells in the ventral cochlear nucleus (VCN) project contralaterally to the medial nucleus of the trapezoid body (MNTB) in the mammalian auditory brainstem. VCN axon terminals in the MNTB form large reticulated structures, known as calyces of Held, which envelop MNTB principle neurons with multiple finger-like extensions [9-11]. This strictly contralateral projection is an essential component of the circuitry that computes interaural time and intensity differences, critical cues for sound source localization [12-16]. In this study we used the VCN-MNTB pathway as a model to study lesion-induced structural plasticity during postnatal development. The projection from VCN to MNTB is well characterized and VCN calyceal terminations in the MNTB are easily identifiable and quantifiable because of their large size. Moreover, because the VCN-MNTB pathway is normally strictly contralateral, lesion-induced aberrant ipsilateral projections are readily observable [17-19].

During early postnatal development in rodents, the survival of VCN neurons is dependent on cochlear input. Removal of the cochlea during the first postnatal week results in substantial VCN cell death [20-22]. Following early unilateral cochlea removal, axons from the intact VCN sprout novel projections directed at sites denervated by the lesion. Notably, under these conditions axons from the intact VCN form calyces in both the ipsilateral and contralateral MNTB [23-25] and the emergence of the ipsilateral projection coincides with maturation of the normal contralateral projection [23,26,27]. In contrast, cochlea removal after the first postnatal week does not induce these novel projections, likely reflecting the survival of VCN neurons after deafferentation at later ages [17,21,22,28,29].

While this lesion-induced sprouting of VCN neurons is observed only when the cochlea is removed before P7, it can be elicited at later ages by direct removal of the VCN. Significant ipsilateral calyceal projections from the intact VCN to the denervated MNTB were observed in gerbils following unilateral cochlear nucleus lesions created at P10 to 15; these induced projections were comparable to those seen with cochlea removal at P2 [17]. Induced ipsilateral projections persisted with P25 lesions, although the number of induced projections declined significantly compared to the earlier ages. These experiments suggest a limited developmental critical period during which intact VCN axons are competent to make new ipsilateral MNTB projections in response to deafferentation of the MNTB. They further demonstrate the formation of new VCN-MNTB projections after the normal contralateral projections are relatively mature. In gerbils, this critical period extends beyond that of deafferentation-induced cell death in the VCN [21,29] and beyond hearing onset.

Factors that regulate the extent of lesion-induced sprouting in the VCN-MNTB pathway and its restriction to a limited postnatal period are incompletely understood. Candidate molecules include the Eph receptor tyrosine kinases and their ephrin ligands, which have significant roles in axon guidance and synapse formation [30,31]. The receptors represent the largest family of receptor tyrosine kinases and are divided into two classes based on sequence homology. EphA receptors bind to ephrin-A ligands, while EphB receptors bind to ephrin-B ligands. While promiscuous binding is seen within classes, limited cross-talk between classes arises from EphA4 binding to ephrin-B ligands [32] and EphB2 binding to ephrin-A5 [33]. Eph/ephrin interactions can promote axon repulsion or attraction, depending on several factors including total concentration [34]. Significantly, the ligands are associated with cell membranes through a glycosyl phosphatidyl inositol linkage (ephrin-A’s) or a transmembrane domain (ephrin-B’s), so that interactions with Eph receptors generally require cell contact. Moreover, these ligands permit reverse signaling, in which Eph receptors elicit cell signaling events in the ephrin-expressing cell [35-37].

Our previous studies suggest that reverse signaling through ephrin-B ligands is a critical factor in limiting VCN inputs to ipsilateral MNTB. Specifically, mutations in *EphB2/B3*, *ephrin-B2*, or *EphA4* result in increased levels of ipsilateral MNTB innervation after cochlea removal during the first few days of postnatal life [19,25]. Here we sought to determine whether these molecules similarly affect the level of VCN-MNTB structural plasticity at later ages following direct removal of the cochlear nucleus. Additionally, we tested whether the function of these molecules affects the length of the developmental critical period for lesion-induced innervation of the ipsilateral MNTB.

In this study we show that mice, like gerbils, show induced projections to the ipsilateral MNTB after cochlear nucleus lesions, but that the critical period for lesion-induced projections is more limited in mice than in gerbils. We found that *EphB2* mutant mice have more induced ipsilateral calyces in the MNTB than wild type mice after unilateral VCN removal at P7, which is similar to their effect after early cochlea removal. Lesions performed at later times showed that wild type mice and mutants had similar critical period length, suggesting that the extent of lesion-induced sprouting and the length of the critical period for this reorganization are regulated by independent factors.

## Results

### EphB2 expression at postnatal ages

Before performing cochlear nucleus removal (CNR), we first tested EphB2 expression in the VCN-MNTB pathway at later postnatal ages, as relative Eph protein expression patterns at the time of lesion formation have been shown to be correlated with lesion-induced reorganization [19,25].

**Figure 1:**
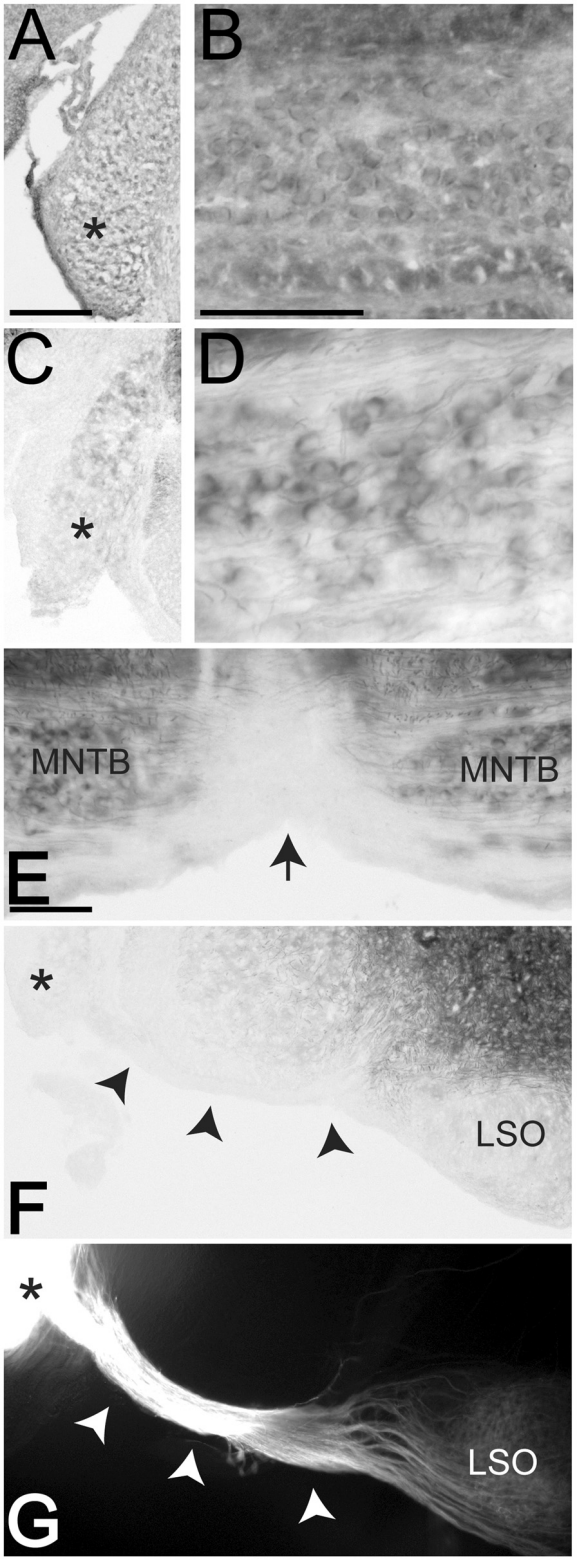
**EphB2 expression at P7.** EphB2 is expressed in the VCN (asterisk, **A**) and in the MNTB (**B**) at P7. Immunohistochemistry results (**A**-**B**) were confirmed with X-gal histochemistry on tissue from P7 *EphB2*^lacZ/lacZ^ mice (**C**-**F**). X-gal staining is present in VCN (asterisk, **C**) and in axons and calyces in MNTB (**D** and **E**). No axonal staining is present at the midline (arrow, **E**), where VCN axons cross to reach the contralateral MNTB. VCN axons in the ventral acoustic stria (arrowheads, **F** and **G**), a population of proximal uncrossed VCN axons, also lack staining (black arrowheads, **F**). Unilateral VCN NeuroVue dye labeling is shown in panel (**G**) to demonstrate the location of the ventral acoustic stria (white arrowheads) in panel (**F**) in relation to VCN (asterisks, **F** and **G**) and lateral superior olive (LSO). Scale bars in panels **B** (also applies to **D**) and **E** represent 100 μm3

At P7, EphB2 was expressed in the VCN (asterisk, Figure 1A) and MNTB (n = 4, Figure 1B), consistent with previously published results [19,38]. We confirmed this pattern of expression using X-gal histochemistry on *EphB2*^lacZ/lacZ^ transgenic mice and found staining similar to the immunohistochemical results (n = 2); staining was observed in the VCN (asterisk, Figure 1C) and in the calyces of Held (Figure 1D). Interestingly, with this assay, in addition to calyceal expression we noted staining in axons near and in the MNTB (Figure 1D-E), but no axonal expression at the midline (Figure 1E), where VCN axons cross to the contralateral MNTB, nor in the VCN axons in the ventral acoustic stria near the VCN (Figure 1F-G), where axons from the VCN on both sides do not cross each other. 

**Figure 2:**
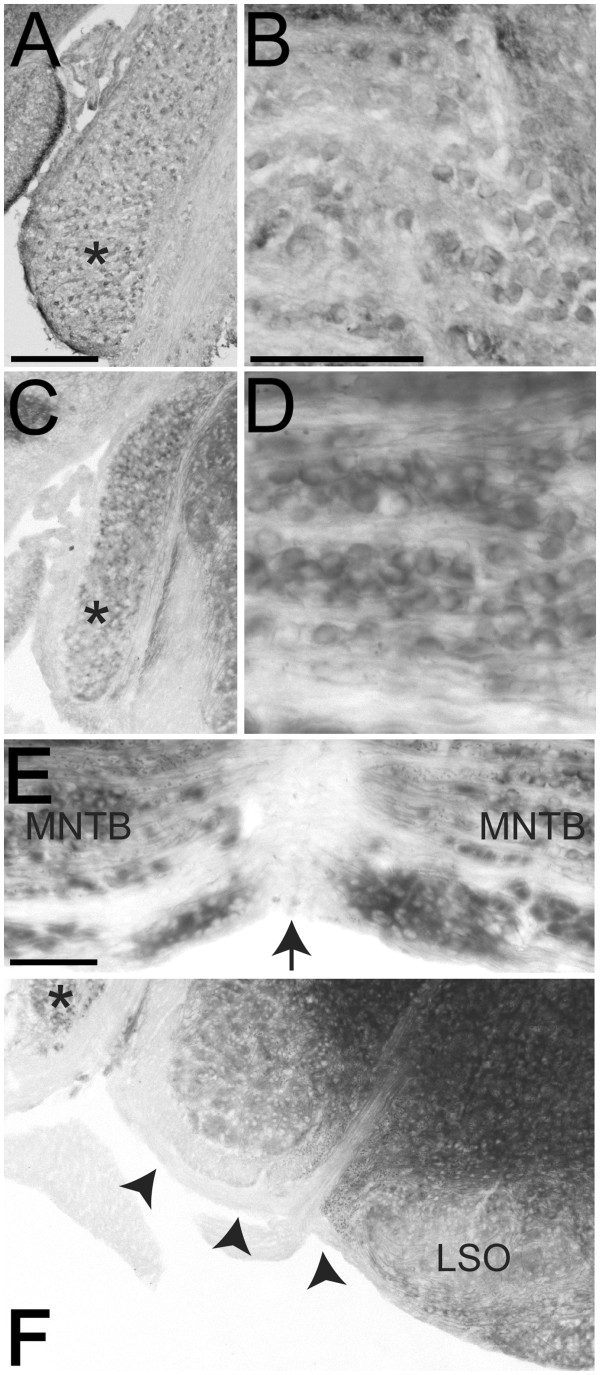
**EphB2 expression at P10.** At P10, EphB2 is expressed in the VCN (asterisk, **A**) and MNTB (**B**). Immunohistochemistry results (**A**-**B**) were confirmed with X-gal histochemistry on tissue from P10 *EphB2*^lacZ/lacZ^ mice (**C**-**F**). Similar to X-gal staining at P7, staining is present in VCN (asterisk, **C**) and in axons and calyces in MNTB (**D** and **E**). No axonal staining is present at the midline (arrow, **E**), where VCN axons cross to reach the contralateral MNTB. VCN axons in the ventral acoustic stria also lack staining (arrowheads, **F**). Scale bar in panel (**A**) represents 200 μm and also applies to panels (**C** and **F**). Scale bars in panels **B** (also applies to **D**) and **E** represent 100 μm.

At P10 we found that EphB2 was expressed in the VCN (asterisk, Figure 2A) and calyces of Held (n = 4, Figure 2B). We also performed X-gal staining on tissue sections from P10 *EphB2*^lacZ/lacZ^ mice to confirm the immunohistochemistry results, and observed a similar staining pattern to the P7 X-gal staining (n = 3). Notably, β-gal was detected in the VCN (asterisk, Figure 2C) and in the calyces of Held (Figure 2D). Additionally, we detected staining in axons in the MNTB (Figure 2D-E), but no axonal expression at the midline (Figure 2E) or in the VCN axons in the ventral acoustic stria ventromedial to the VCN (Figure 2F).

### Cochlear nucleus removal induces VCN projections in mice at P7

**Figure 3:**
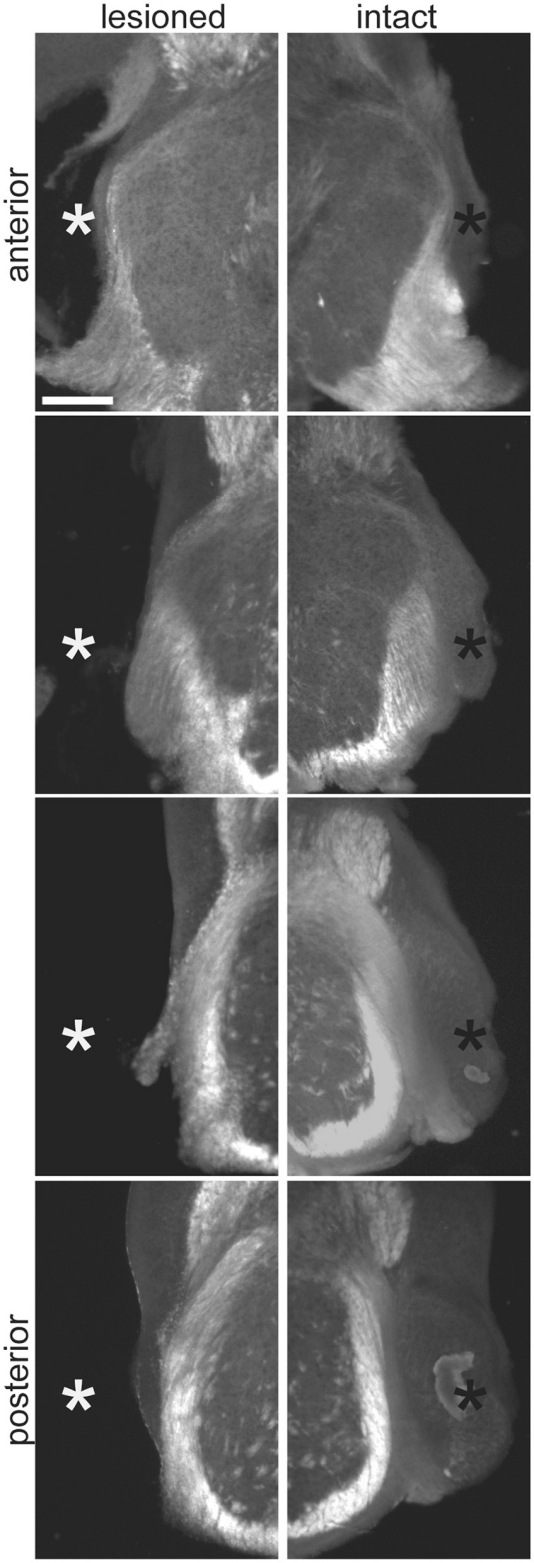
**Unilateral CNR surgery.** Coronal brainstem sections from a mouse with a select and complete unilateral VCN removal (white asterisks). The intact, contralesional VCN is marked by black asterisks. Sections are every 200 μm and the scale bar represents 300 μm.

**Figure 4:**
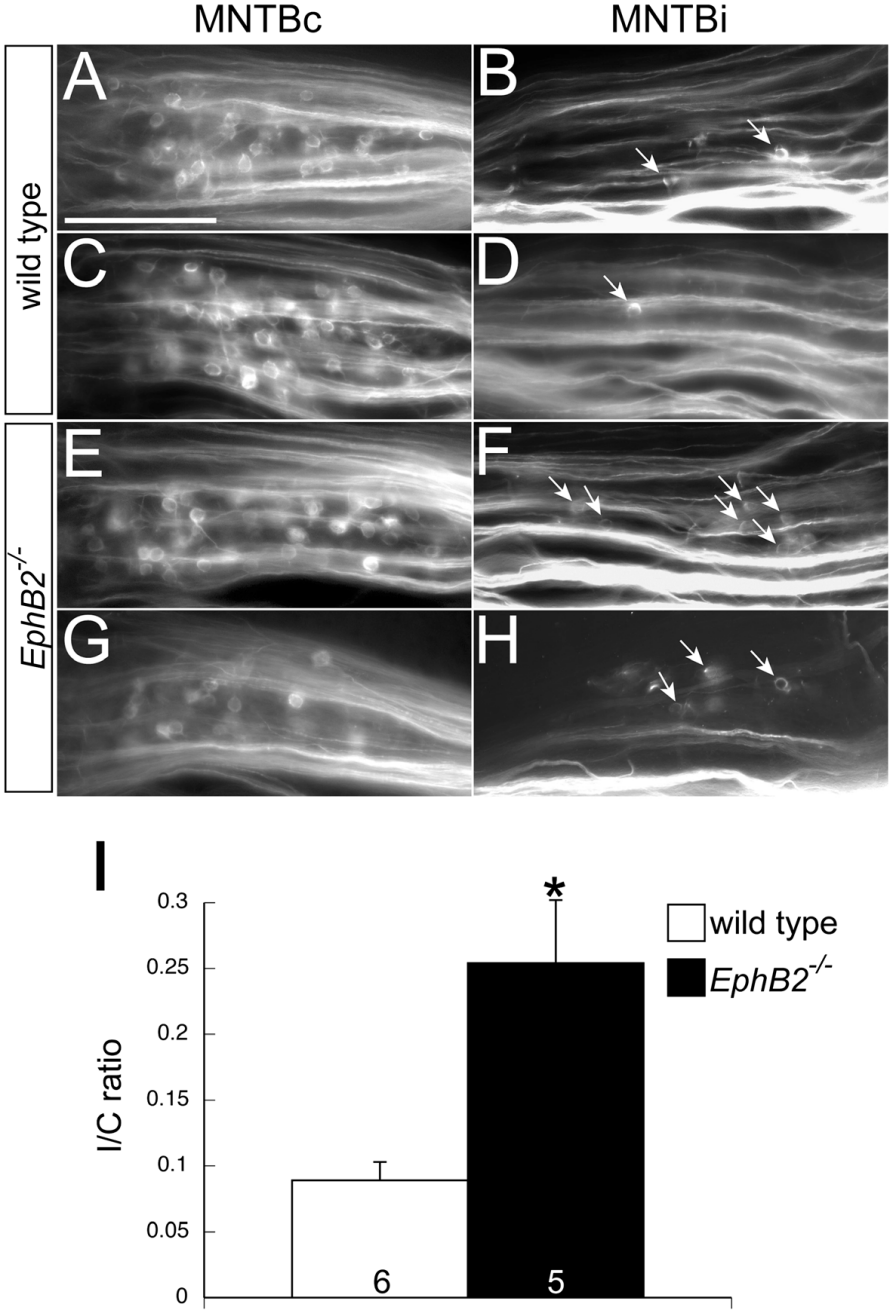
**Unilateral VCN removal at P7 induces ipsilateral projections to the MNTB.** After unilaterally removing the VCN at P7, labeled calyces are seen in the MNTB contralateral (MNTBc) to the intact, dye-filled VCN, as well as in the ipsilateral MNTB (MNTBi, arrows) of wild type (**A**-**D**) and *EphB2*^−/−^ (**E**-**H**) mice. The mean ipsilateral to contralateral (I/C) ratio from *EphB2*^−/−^ mice was greater than the mean I/C ratio from wild type mice (I, *P* < 0.01). Scale bar in panel (**A**) represents 200 μm and also applies to panels (**B**-**H**).

Previous studies showed that CNR results in lesion-induced sprouting of VCN in gerbils [17], but these effects have not previously been documented in mice. We performed CNR in wild type mice at P7. After five to six days, we confirmed the removal of the VCN and labeled axonal projections from the intact VCN (Figure 3). Calyceal terminations were found as expected in the MNTB contralateral to the dye injection (Figure 4A and C). In addition, we observed a significant number of calyceal terminations in the ipsilateral MNTB (Figure 4B and D; Table 1). To obtain a measure of induced ipsilateral projections normalized for variations in dye uptake, we used a ratio of total ipsilateral calyces: total contralateral calyces (I/C ratio). The mean I/C ratio for P7 mice was 0.089 ± 0.014 (n = 6, Figure 4I), which was significantly greater than the mean I/C ratio for unoperated wild type animals of the same age (0.032 ± 0.008; n = 9; *P* < 0.01; data not shown). These data demonstrate that CNR elicits sprouting from intact VCN axons to the ipsilateral, denervated MNTB, and that these sprouts terminate in calyx-like structures.

**Table 1:** Numbers of labeled calyces after cochlear nucleus removal

**Group**	**Ipsilateral (I)**	**Contralateral (C)**	**I/C ratio**
P7 wild type	7	52	0.135
	20	249	0.080
	14	157	0.089
	13	216	0.060
	7	144	0.049
	6	49	0.122
			*Average*: 0.089
P7 *EphB2*^*−/−*^	19	132	0.144
	14	38	0.368
	68	207	0.329
	27	92	0.293
	14	102	0.137
			*Average*: 0.254
P10 wild type	2	138	0.014
	2	47	0.043
	1	143	0.007
	5	236	0.021
	3	276	0.011
			*Average*: 0.019
P10 *EphB2*^*−/−*^	3	39	0.077
	5	125	0.040
	14	240	0.058
	1	154	0.007
			*Average*: 0.045

### EphB2 reduces projections to ipsilateral MNTB after CNR at P7

We previously found that a null mutation in *EphB2* by itself does not affect the targeting of VCN axons to the contralateral versus ipsilateral MNTB during normal development, but it increases the amount of innervation of the ipsilateral MNTB following removal of the cochlea at P2 [19]. To determine if mutations that increase lesion-induced ipsilateral sprouting at early postnatal ages also affect reorganization of this pathway at older ages, when the circuitry is more mature, we performed CNR surgeries in *EphB2*^−/−^ mice at P7. We found that P7 CNR in *EphB2*^−/−^ mice resulted in a mean I/C ratio of 0.254 ± 0.048 (n = 5, Figure 4E-I), which was significantly greater than the I/C ratio obtained from wild type mice subjected to CNR at P7 (*P* < 0.01, Figure 4I) and was comparable to the I/C ratio of wild type mice after P2 cochlea removal [19,25]. The average I/C ratio from the P7 CNR *EphB2*^−/−^ mice was also greater than genotype-matched, non-lesioned animals of the same age that had an average I/C ratio of 0.034 ± 0.011 (n = 7, *P* < 0.01, data not shown). The increased I/C ratio in mutants reflects an increase in induced ipsilateral projections and not a change in contralateral projections, as *EphB2*^−/−^ mice had similar levels of contralateral projections labeled (114.2 ± 24.9; n = 6) as the wild type mice (144.5 ± 34.3; n = 5; *P* = 0.52, *t* test).

### Cochlear nucleus removal at P10 does not elicit ipsilateral VCN-MNTB projections

**Figure 5:**
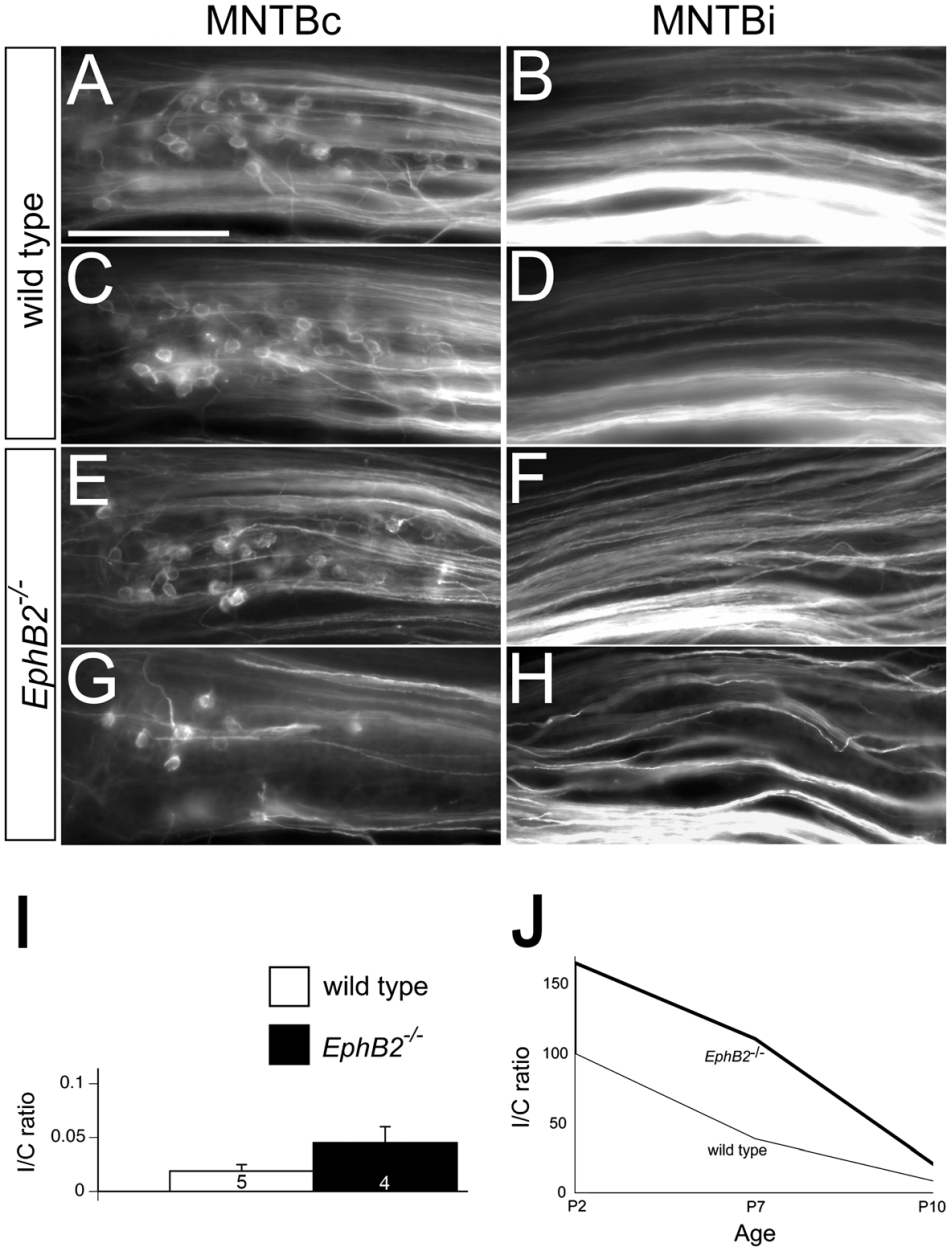
**Unilateral VCN removal at P10 does not induce projections to the ipsilateral MNTB.** Labeled calyces are seen in the contralateral MNTB (MNTBc) of wild type **(A and C)** and *EphB2*^−/−^ mice (**E and G)** after removing the VCN at P10, but not in the ipsilateral MNTB (MNTBi) of either mouse **(B, D, F, H)**. The mean ipsilateral/contralateral (I/C) ratios did not differ after unilateral VCN removal at P10 (**I,***P* > 0.05). Taken together, deafferentation-induced innervation of the ipsilateral MNTB decreases postnatally in wild type and *EphB2*^−/−^ mice **(J)**. *EphB2*^−/−^ mice have more ipsilateral sprouting after unilateral deafferentation at P2 and P7, but not at P10, when the critical period for this process has ended. Y-axis values in panel (**J**) are I/C ratios represented as a percentage of the I/C ratio obtained after cochlea removal in wild type mice at P2. P2 data is from Nakamura *et al*., 2012. Scale bar in panel (**A**) represents 200 μm and also applies to panels (**B**-**H**).

While the critical period for CNR-induced VCN sprouting in gerbils extends beyond hearing onset, the developmental limits in mice have not previously been explored. We found that, as in gerbils, the extent of CNR lesion-induced projections decreases with age. In mice, by P10 this lesion no longer produces significant numbers of new projections. Unlike after P7 CNR, we found very few, if any, labeled calyces in the ipsilateral MNTB of wild type mice after P10 CNR (Figure 5A-D; Table 1). The mean I/C ratio of wild type mice after P10 CNR was 0.019 ± 0.006 (n = 5, Figure 5I) and did not differ (*P* = 0.20) from the P10 sham-operated wild type I/C ratio of 0.010 ± 0.003 (n = 9, data not shown), suggesting no effect of the P10 CNR manipulation in wild type mice. The sharp decline in I/C ratio after CNR was not attributable to differences in the total number of labeled contralateral calyces, which were similar at P10 (168 ± 40.2; *P* = 0.66, *t*-test).

### EphB2 does not increase ipsilateral MNTB innervation after CNR at P10

*EphB2*^*−/−*^ mice showed greatly increased CNR-induced ipsilateral projections after P7 lesions (Figure 4). Here we tested whether this effect persisted at P10, when the critical period appears to have closed in wild type animals, but EphB2 protein is still expressed. Unlike P7 animals, we observed very few ipsilateral calyces in P10 CNR *EphB2*^−/−^mice, with a mean I/C ratio of 0.045 ± 0.015 (n = 4, Figure 5E-I), which did not differ from the mean I/C ratio of wild type mice operated at the same age (*P* = 0.33, Figure 5I), nor from the mean I/C ratio of P10 sham operated *EphB2*^−/−^ mice (0.009 ± 0.005; n = 5; *P* = 0.09, data not shown). At this age, similar to P7, there was no significant difference in the number of contralateral calyces labeled between wild type mice (168 ± 40.2) and *EphB2*^−/−^mice (139.5 ± 41.4; *P* = 0.64, *t* test).

## Discussion

### Species differences in VCN-MNTB developmental plasticity

An unexpected finding of this study was that the window for deafferentation-induced innervation of the ipsilateral MNTB closes between P7 and P10 in mice. This developmental period is early in comparison to the critical period in gerbils, which persists until at least P25 [17]. Species differences may exist in the degree of myelination of VCN axons [39], the maturation of physiological membrane properties of MNTB neurons that may promote calyx formation [40,41], or immunological responses to lesion [42], among other factors. An examination of strain differences in mice with different amounts of lesion-induced sprouting in the VCN-MNTB projection (PAN and KSC, unpublished observations) may provide further insight into this issue [43].

### Development and lesion-induced plasticity

During development of the VCN-MNTB pathway, signaling through ephrin-B ligands in MNTB neurons prevents the formation of calyces in the ipsilateral, but not contralateral, MNTB [38]. We have also previously shown that similar mechanisms are involved in ipsilateral MNTB innervation after unilateral deafferentation at P2. Specifically, mutations that reduce signaling through ephrin-B ligands result in more projections to the ipsilateral MNTB after removal of the cochlea on one side. We have now extended this finding to a later age, when the VCN is directly removed. Together, these data suggest that similar signaling mechanisms are employed during development and during deafferentation-induced rewiring during development.

One possible mechanism by which lesions can induce reorganized projections depends on deafferentation-induced changes in the expression of molecules utilized during normal development. Support for this hypothesis comes from other models of injury. For example, the expression levels of several Eph receptors and ephrins have been shown to change after spinal cord injury [44-48]. Similar changes in Eph protein expression are reported after optic nerve injury [49]. However, these studies may differ from the present study in some important ways. Firstly, spinal cord and optic nerve injuries are performed on adult animals, while we performed our lesions on developing animals. This timing is significant because the expression of Eph proteins in adulthood, at the time of lesion, is different than in the developing system [50]. Secondly, there is very little axonal regeneration after spinal cord and optic nerve injuries, while cochlea or cochlear nucleus removal results in robust axonal sprouting during early postnatal development. These differences highlight some considerable age-related differences in responses to lesion. Thus, a more direct comparison may be an analysis after lesions performed while these circuits are developing.

An alternative view is that the reorganizational process may be guided entirely by the molecular environment at the time of lesion, and not by changes in expression induced by the lesion.

Data to support this possibility come from experiments in *EphA4* mutant mice. EphA4 signaling has no effect on the prevention of calyx formation in the ipsilateral MNTB during development, but dramatically reduces the amount after unilateral deafferentation. Notably, unilateral deafferentation does not induce a change in the level of EphA4 expression in the ipsilateral MNTB [25]. However, these assessments were made immunohistochemically and by comparing the ipsilateral MNTB to the contralateral MNTB of the same animal. A more sensitive and quantitative analysis of gene expression may be necessary, as well as a comparison between operated and non-operated animals, as changes may occur in both MNTBs after lesion. These possibilities are not mutually exclusive, and it is likely that the extent of deafferentation-induced developmental plasticity in the VCN-MNTB projection depends on both current and induced changes in Eph protein expression.

In the results presented here, mutant mice lacking EphB2 have more lesion-induced ipsilateral inputs than wild type animals. While the mechanisms that lead to this difference are not known, some possibilities are related to several functions of EphB2. EphB2 may mediate repulsive effects on VCN axon growth, thus limiting the extent of branching in the denervated MNTB. An alternative possibility, consistent with our previous observations of a non cell-autonomous reverse signaling role, is related to the role of EphB2 in synapse formation [51]. VCN synapses in the MNTB may normally suppress signals that elicit innervation and may be weaker in *EphB2* mutant mice. Weaker synapses are consistent with the observation that *ephrin-B2* mutant mice show reduced activation of c-fos expression in MNTBs compared to wild type mice [52]. MNTB cells that receive weaker innervation may be more prone to eliciting synapses, both during normal development and after denervation. Further studies will be needed to clarify the mechanisms underlying the role of EphB2 in lesion-induced projections.

### Developmental plasticity and critical periods

We have found that EphB2 signaling affects the quantity of structural plasticity in the VCN-MNTB projection after unilateral lesion, but does not affect the closure of the critical period for this event. It is possible that EphB2 signaling does not affect the initial formation of induced collaterals to the ipsilateral MNTB from the contralaterally-projecting axons after removal of the cochlear nucleus [17]. It may be that EphB2 selectively controls the branching of these collaterals, as a single VCN axon can branch and produce several calyces to innervate multiple adjacent MNTB neurons, such that a single VCN axon branches to innervate more ipsilateral MNTB principal neurons in *EphB2* mutant mice than in wild type mice [53,54]. This role in branching is supported by the data presented in this study; we only observed an increase in ipsilateral calyces in *EphB2* mutant mice after P7 when there was some sprouting in wild type mice, but not after P10 CNR, when there was no effect of deafferentation in wild type mice. An analysis of single VCN axons in the ipsilateral MNTB after P7 CNR from wild type and *EphB2* mutant mice would be informative.

Factors that regulate the closure of the critical period in other systems may similarly regulate the decreased sensitivity to changes in input in the VCN-MNTB pathway. In the visual cortex, the maturation of perineuronal nets and inhibitory circuitry has been shown to greatly affect the critical period for plasticity in ocular dominance columns after monocular deprivation [55-57]. Indeed, perineuronal nets are abundant in the MNTB and the expression of chondroitin sulfate proteoglycans, the molecular substrates of these nets, is developmentally regulated [58,59]. Moreover, the maturation of inhibitory circuitry occurs postnatally in the auditory brainstem, as elsewhere in the brain, and in the time frame of the present study [60-62].

## Conclusions

EphB signaling influences the contralateral bias of the VCN-MNTB projection, both during normal development and in lesion-induced plasticity at early postnatal ages, when circuitry is relatively immature [19,38]. In this study we showed that direct removal of the cochlear nucleus elicits projections from the intact VCN to the ipsilateral MNTB in mice at P7, when cochlea removal alone is no longer able to elicit these projections. CNR in mutant mice lacking EphB2 showed that EphB2 signaling also influences the level of ipsilateral MNTB innervation after VCN removal at P7. This finding demonstrates that EphB2 limits ipsilateral VCN-MNTB projections not only during development and after lesions in early development, but also after the normal contralateral calyces have formed. However, while EphB2 mutations greatly increase lesion-induced projections during this critical period, they do not have this effect at later ages and do not extend the critical period. These results suggest that molecular mechanisms that regulate the quantity of lesion-induced ipsilateral projections most likely operate independently from the mechanisms that regulate critical period length for this plasticity (results summarized in Figure 5J).

## Methods

### Animals

All procedures were approved by the University of California, Irvine Institutional Animal Care and Use Committee. Wild type, *EphB2*^null^*,* and *EphB2*^lacZ^*;EphB3*^null^ mice on a CD-1 background were used in this study [63]. Mice were genotyped using PCR on isopropanol precipitated DNA after an intervening lysis step from tail samples as previously described [19,38,63].

### Histochemistry

Wild type mice were euthanized with an overdose of isoflurane and perfused transcardially with 0.9% saline followed by 4% paraformaldehyde in PBS (PFA, pH 7.4). Brainstems were postfixed for two hours at 4°C in PFA and then dehydrated in 30% sucrose in PBS overnight. Coronal sections were cut at 20 μm and directly mounted onto chrome-alum coated slides.

For immunohistochemistry, slides were rinsed with PBS, incubated in 0.1% SDS in PBS for five minutes, rinsed again, and then incubated in 0.03% H_2_O_2_ in methanol for ten minutes. Slides were transferred to tris-buffered saline (TBS) and 0.05% Triton X-100 (TBST), incubated in blocking solution containing 3% normal rabbit serum (Vector Laboratories, Burlingame, CA) in TBST for one hour, and incubated overnight with goat anti-EphB2 polyclonal antibody (R&D Systems, Minneapolis, MN) diluted to 10 μg/mL in 1% normal rabbit serum in TBST. The following day, the slides were rinsed in TBS, incubated in biotinylated rabbit anti-goat secondary antibody (6 μg/mL, Vector Laboratories, Burlingame, CA) diluted in TBST with 1% normal rabbit serum for two hours, rinsed with TBS, and then incubated in ABC solution (Vector Laboratories, Burlingame, CA) for one hour. After rinsing with TBS, slides were reacted with 3,3′-diaminobenzidine (Sigma-Aldrich, St. Louis, MO), rinsed with PBS, dehydrated in a graded series of ethanol to xylenes, and coverslipped with DPX mountant (VWR International, Radnor, PA). Specificity of the anti-EphB2 antibody was confirmed by western blot analysis after immunoprecipitation on brainstem homogenate from wild type and *EphB2*^−/−^ mice, which yielded a single band of 120 to 130 kDA and no band, respectively.

For X-gal histochemistry to visualize β-galactosidase, slides were rinsed in PBS, followed by incubation at 37°C overnight in a solution containing 5 mM potassium ferrocyanide, 5 mM potassium ferricyanide, 2 mM MgCl_2_, and 1 mg/mL 5-bromo-4-chloro-3-inolyl-beta-D-galactopyranoside (Fisher Scientific, Pittsburgh, PA). Slides were rinsed with PBS the following day and coverslipped with Glycergel (Dako, Carpinteria, CA).

### Cochlear nucleus removal

For CNR, the VCN was removed surgically at P7 or P10 as previously described [17]. Briefly, animals were anesthetized by hypothermia (P7) or with ketamine and xylazine (P10; 80 to 100 mg/kg and 1.2 to 2 mg/kg, respectively, intraperitoneally). A small incision was made ventral to the pinna through which the tympanic membrane was exposed and the middle ear mesenchyme and ossicles were aspirated with a sterile pipette. The pipette was then inserted through the oval window and the contents of the cochlea aspirated, followed by removal of the VCN after advancing the pipette a few more millimeters. All procedures were performed under a stereomicroscope using heat-sterilized instruments. Animals were given flunixin (2.5 mg/kg, subcutaneously) immediately after surgery and the following day and were sacrificed five or six days after surgery.

### Neuroanatomical labeling

Mice were euthanized with isoflurane followed by perfusion with 0.9% saline and 4% PFA. Brainstems were removed and postfixed overnight in PFA at 4°C. We labeled the projection from VCN to MNTB with the lipophilic dye NeuroVue Red (PTI Research, Warrington, PA) using a previously described protocol [17,19,25,38]. Briefly, the cerebellum was dissected away and a large piece of dye was placed in the intact VCN which was allowed to transport for two to three weeks in paraformaldehyde (PFA) at 37°C.

### Analysis

Coronal sections were cut at 100 μm on a Vibratome and mounted onto chrome-alum coated slides and coverslipped with Glycergel (Dako, Carpinteria, CA). Sections were imaged using a Zeiss Axioskop microscope, Axiocam camera, and Openlab software (Improvision, Waltham, MA). To compare the amount of lesion-induced sprouting to the ipsilateral MNTB following CNR between genotypes, we calculated an ipsilateral to contralateral (I/C) ratio for each animal to normalize for any inter-animal differences in the amount of dye used [17,19,25,38]. We counted the total number of labeled calyces in the MNTB ipsilateral to the intact, dye-filled VCN and divided this number by the total number of labeled calyces in the contralateral MNTB for each animal. An axonal termination in the MNTB was only counted as a calyx if at least one-third of the MNTB cell surface was covered. All analysis was performed blind to genotype and we only included animals with a complete and selective removal of VCN in our analysis (Figure 3). We used a Wilcoxon-Kruskal-Wallis rank sum test to determine differences in I/C ratios between experimental groups. All values are represented as mean ± the standard error of the mean. Digital images were imported into Adobe Photoshop for brightness and contrast adjustments and then into Adobe Illustrator for photomicrograph construction.

## Abbreviations

CNR: cochlear nucleus removal; I/C: ipsilateral to contralateral ratio; LSO: lateral superior olive; MNTB: medial nucleus of the trapezoid body; P: postnatal day; PBS: phosphate-buffered saline; PCR: polymerase chain reaction; PFA: paraformaldehyde; TBS: tris-buffered saline; VCN: ventral cochlear nucleus.

## Competing interests

The authors declare that they have no competing interests.

## Authors’ contributions

PAN and KSC designed the experiments. PAN performed the experiments and analyzed the data. PAN and KSC wrote the manuscript. Both authors read and approved the final manuscript.
